# Hallucinations in acutely admitted patients with psychosis, and effectiveness of risperidone, olanzapine, quetiapine, and ziprasidone: a pragmatic, randomized study

**DOI:** 10.1186/1471-244X-13-241

**Published:** 2013-09-30

**Authors:** Erik Johnsen, Igne Sinkeviciute, Else-Marie Løberg, Rune A Kroken, Kenneth Hugdahl, Hugo A Jørgensen

**Affiliations:** 1 Division of Psychiatry, Haukeland University Hospital, Sandviken, Norway; 2 Department of Clinical Medicine, Psychiatry, University of Bergen, Bergen, Norway; 3 Department of Biological and Medical Psychology, University of Bergen, Bergen, Norway; 4 Department of Radiology, Haukeland University Hospital, Bergen, Norway

## Abstract

**Background:**

Hallucinations are prevalent in schizophrenia and related psychotic disorders and may have severe consequences for the affected patients. Antipsychotic drug trials that specifically address the anti-hallucinatory effectiveness of the respective drugs in representative samples are rare. The aims of the present study were to investigate the rate and severity of hallucinations in acutely admitted psychotic patients at hospital admission and discharge or after 6 weeks at the latest, if not discharged earlier (discharge/6 weeks); and to compare the anti-hallucinatory effectiveness of risperidone, olanzapine, quetiapine, and ziprasidone with up to 2 years’ follow-up.

**Methods:**

Adult patients acutely admitted to an emergency ward for psychosis were consecutively randomized to risperidone, olanzapine, quetiapine, or ziprasidone and followed for up to 2 years in a pragmatic design. Participants were assessed repeatedly using the hallucinatory behavior item of the Positive and Negative Syndrome Scale (PANSS).

**Results:**

A total of 226 patients, 30.5% of those assessed for eligibility, were randomized and 68% were hallucinating at baseline. This proportion was reduced to 33% at discharge/6 weeks. In the primary analyses based on intention to treat groups of patients experiencing frequent hallucinations, the quetiapine and ziprasidone groups both had faster decreases of the mean hallucination scores than the risperidone group.

**Conclusions:**

Hallucinations are fairly responsive to antipsychotic drug treatment and differential anti-hallucinatory effectiveness may be found among existing antipsychotic drugs. If replicated, this could pave the way for a more targeted pharmacotherapy based on individual symptom profiles, rather than on the diagnostic category.

**Trial registration:**

ClinicalTrials.gov ID; NCT00932529

## Background

Hallucinations, most often auditory in nature, are highly prevalent in schizophrenia and related psychotic disorders but prevalence figures vary greatly among different reports [1]. Auditory hallucinations in schizophrenia can be dramatic and may have severe impact in affected individuals, and are sometimes associated with suicidality [2-4], violence [5,6], and homicide [7]. Accordingly, hallucinations have traditionally been one of the main treatment targets for antipsychotic drugs and indeed the positive psychotic symptoms of schizophrenia collectively are far more responsive to these drugs than negative or other cognitive symptoms [8,9]. Importantly, patients with schizophrenia and related disorders are a very heterogeneous group symptomatically [10]. Consequently, to optimize treatment effectiveness in different symptomatic subgroups of patients, individually tailoring drug regimens should be an important aim. However, the results from clinical drug trials very rarely focus on single symptom scores such as hallucinations but typically report group mean changes of overall psychopathology, or at best the positive subscale scores. In the widely used Positive and Negative Syndrome Scale (PANSS) [11] the hallucination item is one of a total of 7 items in the positive subscale which also includes Delusions, Conceptual disorganization, Excitement, Grandiosity, Suspiciousness, and Hostility. There is an obvious risk that differential anti-hallucinatory effectiveness among antipsychotic drugs may be obscured by means of sum scores for the whole sample in clinical trials.

We have previously reported differential effectiveness for risperidone, olanzapine, quetiapine, and ziprasidone for overall change of the PANSS total and positive subscale scores, respectively [12]. The present study focuses on hallucinations and the hallucinatory behavior (hallucinations) item in the PANSS. The aims of the study were to investigate the rate and severity of hallucinations at hospital admission and discharge, or at 6 weeks from baseline at the latest, if not discharged earlier (discharge/6 weeks), in a large sample of patients acutely admitted with psychosis. Moreover, the study aimed to compare the anti-hallucinatory effectiveness of the second generation antipsychotic drugs (SGAs) risperidone, olanzapine, quetiapine, and ziprasidone with up to 2 years’ follow-up.

## Methods

### Study design

**Figure 1:**
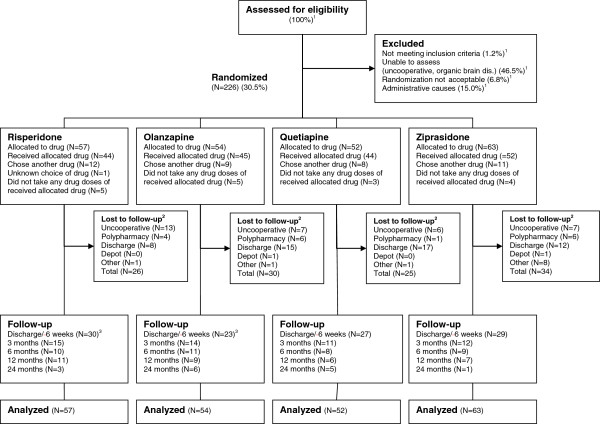
**Flow of patients through the study.** Not meeting inclusion criteria = a score below 4 on all items: Delusions, Hallucinatory Behavior, Grandiosity, Suspiciousness/Persecution, or Unusual thought content in the Positive and Negative Syndrome Scale (PANSS); Uncooperative = the patient was not able or willing to cooperate with testing and assessments; Organic brain dis. = Organic brain disorder, principally dementia; Randomization not acceptable = patient or treating clinician not willing to change existing antipsychotic medication; Administrative causes = principally the result of patient discharge before assessments could be made.^1^ Enrollment started in 2003, week 10 until 2008, week 26. Full details on enrollment were only registered from 2006, week 31 until 2008, week 26. Consequently only percentages are displayed for patients assessed for eligibility and excluded patients.^2^ Before discharge/6 weeks. ^3^ One patient in the risperidone and olanzapine groups missed the first follow-up visit, but was retested on later visits.

The methods used in this study have been described in more detail in a previous publication [12]. The project was a 2-year, prospective, rater-blind, pragmatic, randomized, head-to-head comparison of risperidone, olanzapine, quetiapine, and ziprasidone conducted at Haukeland University Hospital, Bergen, Norway (see Figure 1 for the patient flow in the project).

The project was approved by the Regional Committee for Medical Research Ethics and the Norwegian Social Science Data Services, was publicly funded and did not receive any financial or other support from the pharmaceutical industry.

### Patients

The Regional Committee for Medical Research Ethics allowed eligible patients to be included before informed consent was provided, thus entailing a clinically relevant representation in the study.

#### *Inclusion criteria*

Adult patients were eligible for the study if they were admitted to the emergency ward for symptoms of active psychosis as determined by a score of ≥ 4 on one or more of the items Delusions, Hallucinatory behavior, Grandiosity, Suspiciousness/persecution, or Unusual thought content in the PANSS [11], and were candidates for oral antipsychotic drug therapy with one of the study drugs. Importantly, the study drugs represented the available first-choice SGAs in Norway for psychosis at the time of the conductance of the study. All eligible patients met the ICD-10 (http://apps.who.int/classifications/icd10/browse/2010/en) diagnostic criteria for schizophrenia, schizoaffective disorder, acute and transient psychotic disorder, delusional disorder, drug-induced psychosis, bipolar disorder except manic psychosis, or major depressive disorder with psychotic features. The diagnoses were determined by the hospital’s psychiatrists or specialists in clinical psychology.

#### *Exclusion criteria*

Patients were excluded from the study if they were unable to use oral antipsychotics, were suffering from manic psychosis or for other behavioral or mental reasons related to the state of illness were unable to cooperate with assessments, did not understand spoken Norwegian, were candidates for electroconvulsive therapy, or were medicated with clozapine on admittance. Patients with drug-induced psychoses were included only when the condition did not resolve within a few days and when antipsychotic drug therapy was indicated.

### Treatments

At admission, a sealed and numbered envelope was opened by the treating psychiatrist or physician and then the patient was offered the first drug in a random sequence of risperidone, olanzapine, quetiapine, or ziprasidone. The randomization was open to the treating psychiatrist or physician and to the patient, and either party could discard the SGA listed as #1 on the list because of medical contraindications for the use of, or prior negative experiences with the drug, and the next drug on the list could be chosen. The same principle was followed if the next drug could not be used. The protocol thus mimicked the usual clinical situation in which oral antipsychotic drug therapy is initiated: To prospectively predict which antipsychotic might be optimal for a given patient is not possible based on the current evidence [13], but a prior history of antipsychotic drug use may provide useful information for decision making. A reason for discarding drugs should be explicitly stated by the treating psychiatrist or physician. The SGA listed as #1 defined the randomization group (RG) which is the basis of the primary analysis. The actual SGA chosen, regardless of randomization group, defined the first-choice group (FCG). Further dosage, combinations with other drugs, or switching to another antipsychotic drug were then left at the treating clinician’s discretion. Apart from sporadic use, the patients in the project could use only one antipsychotic drug except during the cross-titration period during a change in antipsychotic drug treatment.

### Clinical assessments

Study visits by the patients were at baseline, at discharge or at 6 weeks from baseline at the latest if not discharged earlier (discharge/6 weeks), and at 3, 6, 12, and 24 months from baseline. The patients were not excluded if they changed their antipsychotic medication during the inclusion period, but importantly only data obtained during the use of the first antipsychotic drug have been included in the present study.

#### *Baseline*

Before inclusion, eligible patients underwent the PANSS structured clinical interview. Intra-class correlation coefficients (ICC) were calculated based on inter-rater assessments and showed high inter-rater reliability (0.92). Furthermore, the patients underwent assessments using the Calgary Depression Scale for Schizophrenia (CDSS) [14], and the Clinical Drug and Alcohol Use Scales (CDUS/CAUS) [15], as well as a neurocognitive test battery, and were rated according to the Clinical Global Impression—Severity of Illness scale (CGI-S) [16], and the Global Assessment of Functioning—Split Version, Functions scale (GAF-F) [17].

#### *Discharge/ 6 weeks*

At discharge/6 weeks, the tests and examinations were repeated by raters unaware of which treatment the patient was receiving. Serum level concentration of the antipsychotic drug was measured. All of these tests and measurements were part of the hospital’s routine procedure for the handling and management of patients suffering from psychosis, and was included in the patient’s medical record. At this point in the study procedure, the patients were asked for written informed consent to be later contacted and asked to participate in the follow-up project.

#### *Subsequent assessments*

At follow-up visits 3, 6, 12, and 24 months after baseline the same measures and tests were repeated, and all medication use was recorded. Doses for the accepted sporadic use of antipsychotics other than the SGAs under investigation were converted to chlorpromazine equivalent doses [18]. In cases where chlorpromazine equivalent doses could not be found in the literature, this was done by conversion to defined daily doses (DDDs) as developed by the World Health Organization Collaborating Center for Drug Statistics Methodology (http://www.whocc.no/atcddd/).

### Statistical procedures

Categorical and continuous data at baseline and at discharge/6 weeks, respectively, were analyzed by means of exact χ^2^ - tests and one-way ANOVAs by using the SPSS software, version 20.0 (IBM SPSS Statistics, 2011). For comparing mean hallucination scores at baseline and at discharge/6 weeks, paired-sample t-tests were used. For baseline comparisons between the patients lost to follow-up before retesting and the patients who were retested, independent-sample t-tests were used for continuous data and exact χ^2^ - tests for categorical data. Comparisons of the time until discontinuation of the individual drug were analyzed by means of Kaplan-Meier survival analyses. Patients with a score of 3 or more on the hallucinations item in the PANSS, hereafter termed the Hallucinatory Patients (HP) subgroup, were followed longitudinally over the measurement period of up to 2 years. The primary analyses were intention-to-treat (ITT) analyses based on the randomization groups (RGs); that is, trial participants were analyzed in the group to which they were randomized regardless of which antipsychotic treatment they actually received. This is in accordance with the recommendations of the Cochrane Handbook for Systematic Reviews of Interventions [19]. The analyses based on the actual SGA chosen at baseline, the first-choice groups (FCGs), were considered secondary analyses as these groups were not based on randomization but rather on the active choice of the patient or treating psychiatrist/ physician. Change of the PANSS hallucinations item score was analyzed in R by means of a linear mixed-effects (LME) model (http://www.r-project.org) [20]. Fixed effects, i.e. systematic differences between the drugs, gave different linear slopes in the four treatment groups, technically a group by time interaction with no baseline group differences. The model calculated overall change per time unit for the variables in the follow-up period that could be visually represented by the slope of a linear curve with time on the x-axis and the hallucination score on the y-axis. For multiple comparisons, Benjamini-Hochberg adjustments were applied. The level of statistical significance was set at α = 0.05, two-sided.

#### *Power analyses*

Power estimations were conducted in R by means of LME models. The baseline mean hallucinations item score and standard deviations (SD) were based on the results of the model used, and slopes corresponding to what was considered the least clinically significant difference among the drug groups were entered into the model. An estimated dropout rate of 3% per month was used and 10,000 simulations were run. The power analyses revealed that with 37 subjects in each treatment arm the trial had 96% power to detect 12.5% differences in the change of the hallucinations item score among the drug groups, whereas the trial had 81% power to detect 10% differences. Smaller differences had inferior power but were not considered clinically significant.

## Results

### The total sample

**Figure 2:**
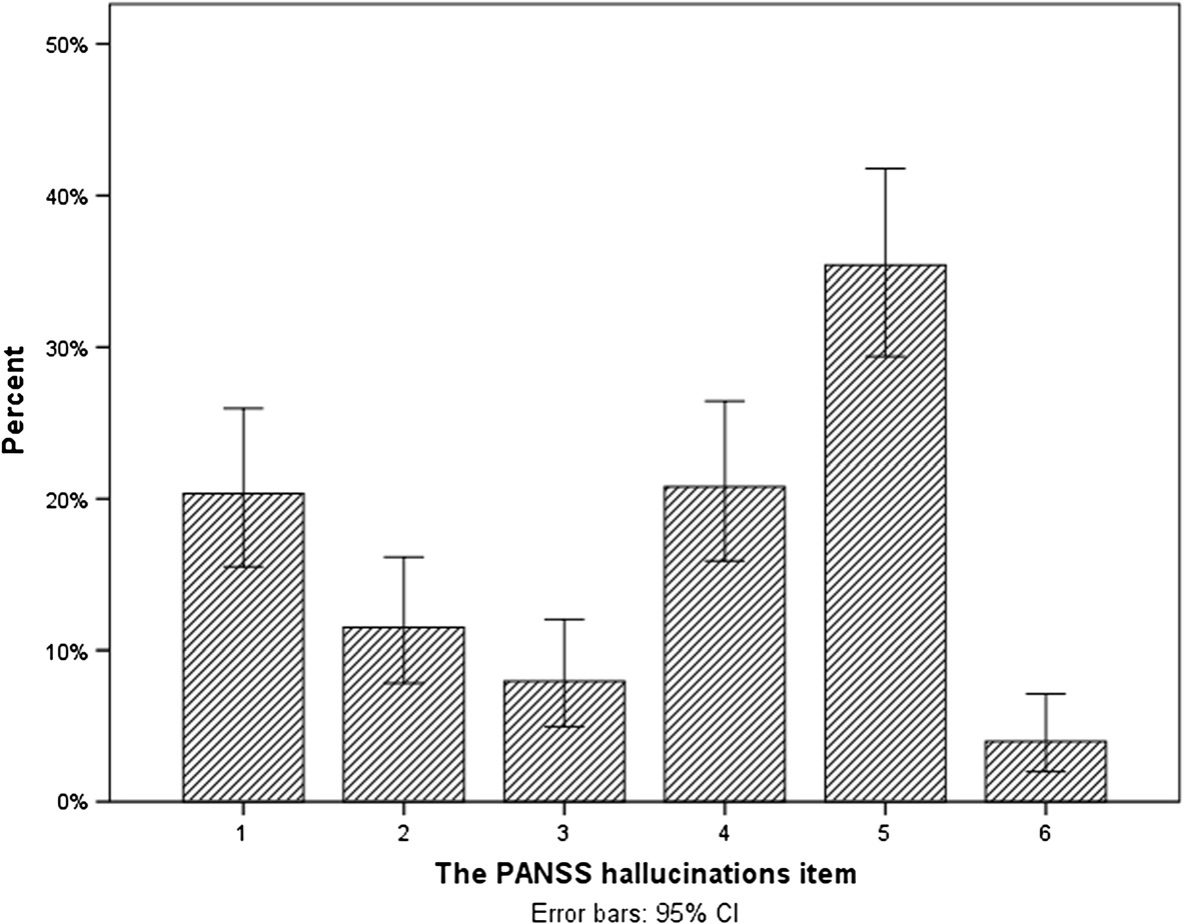
Dispersion of baseline scores of the PANSS hallucinations item.

A total of 226 patients, 152 (67.3%) males, were included and randomized to one of the study drugs (Figure 1). The mean age was 34.1 years, the standard deviation (SD) 13.5. About half the sample was antipsychotic drug naïve at inclusion meaning no prior exposure to antipsychotic agents, whereas the rest had a life-time exposure to antipsychotic drugs. The mean PANSS total score at baseline was 74.0 (13.4), range 44–111, and the global neurocognitive functioning t-score was 38.2 (7.7). A total of 154 (68.1%) patients had a PANSS hallucinations item (P3) score of 3 or more at baseline (the Hallucinating Patients (HP) subgroup); see dispersion of ratings in Figure 2.

The mean and median time periods between baseline and discharge/6 weeks were 28.5 and 28.0 days, SD 14.2, respectively. The proportion with hallucinations was 33.0% at discharge/6 weeks. The reduction of the proportion with hallucinations was statistically significant (Exact χ^2^- test: p = 0.014). The mean hallucinations score was reduced from 3.6 at baseline to 2.0 at discharge/6 weeks (paired-samples t-test: p < 0.001; mean difference 1.6; 95% CI 1.2-1.9).

### The sub-sample with hallucinations

In the HP subgroup that was followed for up to 2 years, the dispersion of patients among the randomization groups was 37, 37, 38, and 42 for risperidone, olanzapine, quetiapine, and ziprasidone, respectively. Demographics and clinical characteristics of the HP subgroup are displayed in Table 1, with no differences among the groups.

**Table 1:** Baseline demographics and clinical characteristics of hallucinations group

		**Randomization groups**									
**Characteristics**		**Risperidone (N = 37)**		**Olanzapine (N = 37)**		**Quetiapine (N = 38)**		**Ziprasidone (N = 42)**		**All patients (N = 154)**	
		**N**	**%**	**N**	**%**	**N**	**%**	**N**	**%**	**N**	**%**
**Gender**											
	**Male**	28	75.7	24	64.9	24	63.2	25	59.5	101	65.6
**Antipsychotic naïve**		13	36.1	14	37.8	21	55.3	22	52.4	70	45.8
**Alcohol last 6 months**											
	**None**	11	29.7	6	16.2	5	13.2	8	19.5	30	19.6
	**Misuse**	3	8.1	4	10.8	9	23.7	4	9.8	20	13.1
**Drugs last 6 months**											
	**None**	19	52.8	26	70.3	28	73.7	26	63.4	99	65.1
	**Misuse**	11	30.6	6	16.2	5	13.1	9	21.9	31	20.4
**Diagnosis**^**1**^											
	**Schz and rel.**	18	52.9	16	44.5	24	63.1	20	51.3	78	53.1
	**Acute**	2	5.9	8	22.2	3	7.9	4	10.3	17	11.6
	**Drug-induced**	8	23.5	4	11.1	6	15.8	5	12.8	23	15.6
	**Affective**	3	8.8	4	11.1	3	7.9	3	7.7	13	8.8
	**Rest**	3	8.7	4	11.1	2	5.2	7	18.0	16	11.0
		**Mean**	**SD**	**Mean**	**SD/Range**	**Mean**	**SD/Range**	**Mean**	**SD/Range**	**Mean**	**SD**
**Age**		32.9	12.0	30.1	11.9	35.8	12.8	29.1	11.5	31.9	12.2
**PANSS Total**		76.8	13.5	77.2	14.2	76.2	14.6	74.4	12.8	76.1	13.7
**PANSS Positive**		20.2	4.6	21.7	4.6	21.0	4.2	20.4	4.6	20.8	4.5
**PANSS Negative**		21.7	8.0	18.7	7.9	19.8	7.2	19.0	7.0	19.8	7.5
**PANSS General**		34.8	6.5	36.9	7.8	35.3	7.3	35.0	6.7	35.5	7.1
**CDSS**		7.2	5.8	7.0	4.5	7.1	4.6	7.6	6.0	7.2	5.2
**GAF-F**		31.4	5.3	29.3	5.6	30.6	7.3	29.9	6.7	30.3	6.3
**CGI**		5.3	0.6	5.3	0.7	5.3	0.6	5.1	0.5	5.3	0.6

***Notes:*** N = number of patients; SD = standard deviation; Antipsychotic naïve = No lifetime exposure to antipsychotic drugs before index admission; First admission = Index admission was the first admission to a mental hospital; Misuse = Misuse or dependence according to Drake et al. [15]; Schz and rel. = Schizophrenia and related disorders: Schizophrenia, schizoaffective disorder, acute polymorphic psychotic disorder with symptoms of schizophrenia, acute schizophrenia-like psychotic disorder, delusional disorder; Acute = Acute psychosis other than those categorized under Schz and rel.; Affective = Affective psychosis; Rest = Miscellaneous psychotic disorders. All diagnoses are according to ICD-10; PANSS = the Positive and Negative Syndrome Scale; CDSS = the Calgary Depression Scale for Schizophrenia; GAF-F = the Global Assessment of Functioning, Split Version, Functions scale; CGI = the Clinical Global Impression, Severity of Illness Scale.

^1^ Patients with missing diagnoses are not included in the list.

There were no differences between those patients lost to follow-up before retesting and those who were retested on clinical and demographic characteristics. A total of 78.4% of the patients chose the #1 drug on the list. There were no significant differences between the randomization groups for the proportion that accepted the #1 drug, nor was there any difference for the choice of actual drug. The mean daily doses, with SD, were 3.4 (1.2) mg, 14.8 (5.4) mg, 325.9 (185.8) mg, and 100.9 (46.7) mg for the risperidone, olanzapine, quetiapine, and ziprasidone groups, respectively. The mean serum levels in nanomoles per liter with SD and reference ranges in brackets were 68.5 (56.2) [30–120], 90.3 (64.4) [30–200], 321.5 (407.3) [100–800], and 107.2 (73.1) [30–200] for risperidone, olanzapine, quetiapine, and ziprasidone, respectively. There were no significant differences between the groups with regards to the use of concomitant psychotropics such as the sporadic use of another antipsychotic drug (N = 11); concomitant antidepressants (N = 19); mood stabilizers (N = 5); benzodiazepines (N = 15); or anti-cholinergic drugs (N = 9) during follow-up. Neither were there any significant differences between the drugs with regards to the use of antipsychotics or type of antipsychotic drugs in the year prior to inclusion in the study. The mean and median time until discontinuation of the randomization drugs with standard error (SE) were 369.6 (72.4) days and 77.0 (26.7) days, respectively, with no significant differences between the randomization groups.

### The primary LME analyses

**Figure 3:**
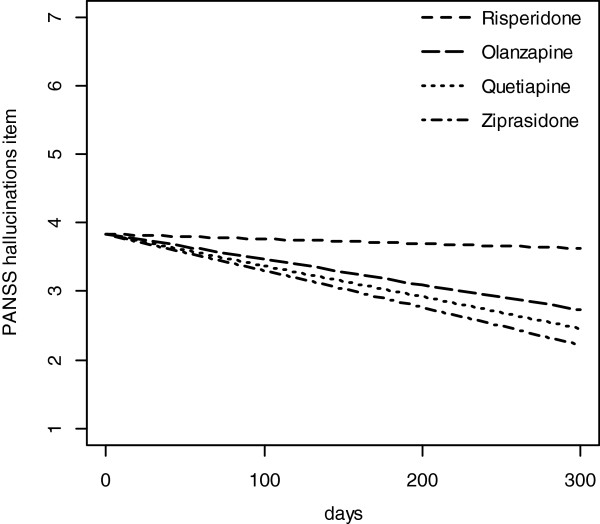
**Reduction of the mean hallucinations score.** Linear slopes for the randomization groups generated based on linear mixed-effects models for the PANSS hallucinatory behavior item. The curves are confined to the first 300 days because the major bulk of data is obtained before 300 days. PANSS = the Positive and Negative Syndrome Scale.

In the primary LME analyses that were intention-to-treat analyses based on the randomization groups, the slopes with standard error (SE) were -0.0007 (0.0013), -0.0037 (0.0010), -0.0046 (0.0011), and -0.0054 (0.0015) for risperidone, olanzapine, quetiapine, and ziprasidone, respectively (Figure 3). The quetiapine and ziprasidone groups had significantly steeper slopes than the risperidone group (LME: p = .027, adjusted for multiple comparisons). The distribution of slopes among the groups remained unaltered in the sensitivity analyses also after adjusting for numerically more drug-naïve patients in the quetiapine and ziprasidone groups. The distribution of slopes for the groups remained unaltered (-0.0020 (0.0018), -0.0036 (0.0011), -0.0047 (0.0011), and -0.0061 ((0.0025)) for risperidone, olanzapine, quetiapine, and ziprasidone, respectively, although the difference did not reach statistical significance, even when patients that did not accept the #1 drug on the list were excluded.

### The secondary LME analyses

In the secondary analyses based on first-choice groups there were no significant baseline differences among the groups with regards to clinical characteristics and demographics except for a higher mean PANSS positive subscale score in the olanzapine group (22.5 points) than in the ziprasidone group (19.8 points) (one-way ANOVA: p = 0.032; mean difference 2.7 points; 95% CI 0.15-5.3). In the LME analyses there were no significant differences among the groups.

## Discussion

The main findings of the present study are that in a consecutive sample of patients acutely admitted to hospital with symptoms of psychosis more than two-thirds had hallucinations at admittance whereas only one-third was hallucinating at discharge/6 weeks. The mean PANSS hallucination score also declined significantly. A substantial reduction of frequency and severity of hallucinations can thus be expected in about half the cases during the first month after hospital admittance. Moreover, differential anti-hallucinatory effectiveness was found in the primary intention-to-treat analyses among the drugs since ziprasidone and quetiapine were both superior to risperidone in reducing the hallucination score, with olanzapine in an intermediate position, when the patients were followed for up to 2 years. The finding is somewhat unexpected as olanzapine and risperidone generally are found to be slightly superior to some of the other SGAs in recent meta-analyses of overall antipsychotic efficacy [21,22]. The samples of most other randomized controlled antipsychotic drug trials are however highly selected [23]. Our sample is more heterogeneous as reflected by the diagnostic diversity and relatively low mean PANSS scores with substantial inter-individual variation, which may explain the apparent discrepancy of the findings between our study and others. For comparison, Sommer et al. [24] recently published outcomes for hallucinations from the EUFEST study comparing the effectiveness of haloperidol, olanzapine, amisulpride, quetiapine, and ziprasidone in patients with first-episode schizophrenia. In their total sample of 498 patients, 73% had scores of at least 3 on the PANSS hallucinatory behavior item, which is a comparable proportion to the 68% in our study. The EUFEST subgroup with a score of at least 3 on the PANSS hallucinations item had a mean PANSS hallucinations score reduction from 4.4 to 2.5 during the first four weeks of treatment, and a further reduction to 1.5 after 6 months [24]. Only 8% had hallucinations after 12 months. The corresponding reduction in our study from baseline to maximally 6 weeks was from 3.6 to 2.0 points. One might expect that symptom reduction would be slower in our more heterogeneous sample as response to antipsychotics is generally best in first-episode psychosis patients, with increasing duration of psychotic symptoms with each relapse [25,26]. At least in the acute phase, no apparent differences in overall reduction of hallucinations were evident between the EUFEST sample [24] and our sample. With regards to comparisons between the different antipsychotic drugs, haloperidol had the least steep reduction of hallucinations for the groups included in the EUFEST sample, and the difference was largest compared to olanzapine, although not statistically significant after adjusting for multiple comparisons [24]. The trend for superiority of olanzapine in the EUFEST sample is, according to the previous discussion, as expected, whereas the finding of superiority for ziprasidone and quetiapine in our sample is more surprising. Based on visual inspection of the steepness of the growth curves for the individual drugs in the EUFEST, ziprasidone and quetiapine seem to be in intermediate positions among the drugs with regards to mean decrease in hallucination severity [24]. This discrepancy in results may indicate that our findings regarding ziprasidone and quetiapine should be interpreted with caution until replicated. The inferiority of haloperidol in its effect on hallucinations is intriguing in relation to our finding of risperidone as having the least steep reduction for the hallucination score. All currently available antipsychotic drugs antagonize dopaminergic transmission at the dopamine type 2 (D2) receptor, while SGAs are characterized by a lower affinity for the D2 receptor than the first-generation drugs, combined with a relatively stronger serotonergic antagonism at the 5HT2A receptor [27,28]. Pharmacologically, risperidone is the SGA that resembles haloperidol closest with regards to D2 receptor affinity, both drugs being very potent D2 receptor antagonists [29,30]. Antipsychotic drug D2 receptor occupancy of 65-70% has been proposed as the optimal therapeutic window for antipsychotic efficacy [31]. However, accumulating evidence indicates a more complex interplay with the dopaminergic system and that additional mechanisms beyond D2 antagonism are at work mediating the antipsychotic drug effects. It was recently demonstrated in a meta-analysis that D2 receptor occupancy accounts for less than 20% of the clinical response variance [32]. Both quetiapine and the SGA prototype – clozapine – have low affinities for the D2 receptor and do not reach receptor occupancies corresponding to the therapeutic window at clinical doses [31,32]. The inferiority of haloperidol and risperidone found in the EUFEST sample and our sample, respectively, could thus be interpreted as additional support that potent D2 receptor antagonism is not the only mediator of anti-hallucinatory drug effects. With regards to alternative biological substrates of drug effects, a striking feature is the pharmacological heterogeneity of antipsychotics [30,33]. Moreover, there is emerging evidence from both clinical and preclinical studies suggesting differential effects among the antipsychotic drugs on non-dopaminergic, non-serotonergic drug targets suggesting that differential drug effectiveness is to be expected in clinical samples with schizophrenia [34-40]. The general lack of finding robust differences for antipsychotics may also be attributed to methodological issues, as pointed out by Leucht and collaborators [23]. An advantage of the present study is the use of the pragmatic, randomized design, emphasizing more representative samples and treatment settings than in traditional RCTs of efficacy, among others [41]. A different aspect is that the vast majority of studies report sum scores. However, idiosyncratic symptom profiles from different sub-samples may level each other out in the data of the collected sample. Another advantage of the present study is the specific focus on hallucinations. To the best of our knowledge, clinical antipsychotic drug trials very rarely study drug effects on single psychotic symptoms, and the current results should encourage an increase in more symptom-based studies, which could be extended to other domains, such as gene-screening studies. The present study focuses exclusively on data obtained during the period of actual use of the drugs studied, which should increase confidence in the findings of differential effectiveness. The results are further strengthened by the serum level measurements which revealed serum drug levels within the reference ranges for all the comparators.

Some limitations should be mentioned. The pragmatic design allows for broader inclusion criteria and fewer exclusion criteria than for traditional RCTs of efficacy, but still only 30% of those assessed for eligibility were included in the study. Although this proportion is at the higher end compared to many antipsychotic drug trials, which may include as little as 10% of the population under investigation [23], there is a risk of selection bias. The direction of the influence of any selection bias related to our results is hard to predict. Another concern is the high attrition rate in our study. Attrition is a major problem in all antipsychotic drug studies and can exceed 40% in studies of only 4 to 10 weeks’ duration [23]. Total attrition was, however, not related to any baseline clinical or demographical characteristics and there were no differences among the randomization groups with regards to mean duration of treatment. The randomization was open to both the attending clinicians and the patients in order to mimic usual clinical practice, but this could theoretically have introduced bias if there were systematic utilization differences among the drugs before the start of the study and some of the SGAs under investigation were associated with more prior experience than the others or were more popular among the clinicians or patients. The direction of such theoretical bias is hard to predict, as both negative and positive prior experiences could influence the attitude towards the SGAs under investigation. There were no substantial differences between the randomization groups with regards to the agents used in the 12 months prior to inclusion or in the proportion who accepted the first SGA on the list. About half the sample had life-time exposure to antipsychotic drugs at study inclusion but noncompliance is a common problem in this patient group and a frequent cause of relapse [42], and most likely only a minority had used antipsychotic drugs according to their prescriptions in the last period of time before admittance. Serum drug levels were not measured at admittance, so the exact figures cannot be verified. The secondary analyses based on the actually chosen drugs failed to find statistically significant differences among the groups.

Our primary analyses were, however, intention-to-treat analyses based on the randomization groups. The secondary analyses do not take advantage of the randomization and are accordingly vulnerable to confounding factors which could have biased the results. There were indeed statistically significant differences among the first-choice groups on the PANSS positive subscale score.

## Conclusions

Despite the limitations, the study suggests that differential anti-hallucinatory effectiveness may exist between risperidone, olanzapine, quetiapine, and ziprasidone, but our study results needs replication before any recommendations for specific drugs for hallucinating patients can be made. The biological substrates mediating any potential differential drug effects on hallucinations remain largely unknown, but future studies with translational designs should address this important issue to pave the way for a more targeted pharmacotherapy of psychosis.

## Competing interests

Erik Johnsen has received honoraria for lectures given in meetings arranged by Bristol-Myers Squibb, Eli Lilly, and AstraZeneca, and for a contribution to an information brochure by Eli Lilly. Erik Johnsen has been reimbursed by the Eli Lilly company and the Janssen-Cilag company for attending conferences.

Igne Sinkeviciute has been reimbursed by the Pfizer company for attending a conference.

Rune Kroken has been reimbursed by the Eli Lilly company, the Janssen-Cilag company, Bristol-Myers Squibb and AstraZeneca for attending conferences.

Else-Marie Løberg declares no competing interests.

Kenneth Hugdahl declares no competing interests.

Hugo A. Jørgensen has been reimbursed by the Eli Lilly company for a contribution to an information brochure.

## Authors’ contributions

All authors made substantive intellectual contributions to the study. EJ collected data, undertook the statistical analyses, and wrote the first draft of the manuscript; IS contributed in analyses and interpretations of the data, and helped draft the manuscript; RAK collected data, contributed in analyses and interpretations of the data, and helped draft the manuscript; E-ML collected data, contributed in analyses and interpretations of the data, and helped draft the manuscript; KH contributed in analyses and interpretations of the data, and helped draft the manuscript; HAJ designed the study, assisted in data collection, contributed in analyses and interpretations of the data, and contributed to the drafting of the manuscript. All authors have read and given final approval of the latest version of the manuscript.

## Pre-publication history

The pre-publication history for this paper can be accessed here:

http://www.biomedcentral.com/1471-244X/13/241/prepub
